# Green and White Asparagus *(Asparagus officinalis)*: A Source of Developmental, Chemical and Urinary Intrigue

**DOI:** 10.3390/metabo10010017

**Published:** 2019-12-25

**Authors:** Eirini Pegiou, Roland Mumm, Parag Acharya, Ric C. H. de Vos, Robert D. Hall

**Affiliations:** 1 Laboratory of Plant Physiology, Wageningen University & Research, P.O. Box 16, 6700AA Wageningen, The Netherlands; eirini.pegiou@wur.nl; 2 Business Unit Bioscience, Wageningen University & Research, P.O. Box 16, 6700AA Wageningen, The Netherlands; roland.mumm@wur.nl (R.M.); ric.devos@wur.nl (R.C.H.d.V.); 3 Unilever Foods Innovation Centre, Bronland 14, 6708WH Wageningen, The Netherlands; parag.acharya@unilever.com; 4 Netherlands Metabolomics Centre, Einsteinweg 55, 2333CC Leiden, The Netherlands

**Keywords:** asparagus, secondary metabolites, asparagus aroma, flavour, phytonutrients, plant metabolomics

## Abstract

Asparagus *(Asparagus officinalis)* is one of the world’s top 20 vegetable crops. Both green and white shoots (spears) are produced; the latter being harvested before becoming exposed to light. The crop is grown in nearly all areas of the world, with the largest production regions being China, Western Europe, North America and Peru. Successful production demands high farmer input and specific environmental conditions and cultivation practices. *Asparagus* materials have also been used for centuries as herbal medicine. Despite this widespread cultivation and consumption, we still know relatively little about the biochemistry of this crop and how this relates to the nutritional, flavour, and neutra-pharmaceutical properties of the materials used. To date, no-one has directly compared the contrasting compositions of the green and white crops. In this short review, we have summarised most of the literature to illustrate the chemical richness of the crop and how this might relate to key quality parameters. Asparagus has excellent nutritional properties and its flavour/fragrance is attributed to a set of volatile components including pyrazines and sulphur-containing compounds. More detailed research, however, is needed and we propose that (untargeted) metabolomics should have a more prominent role to play in these investigations.

## 1. The Asparagus Crop

**Figure 1:**
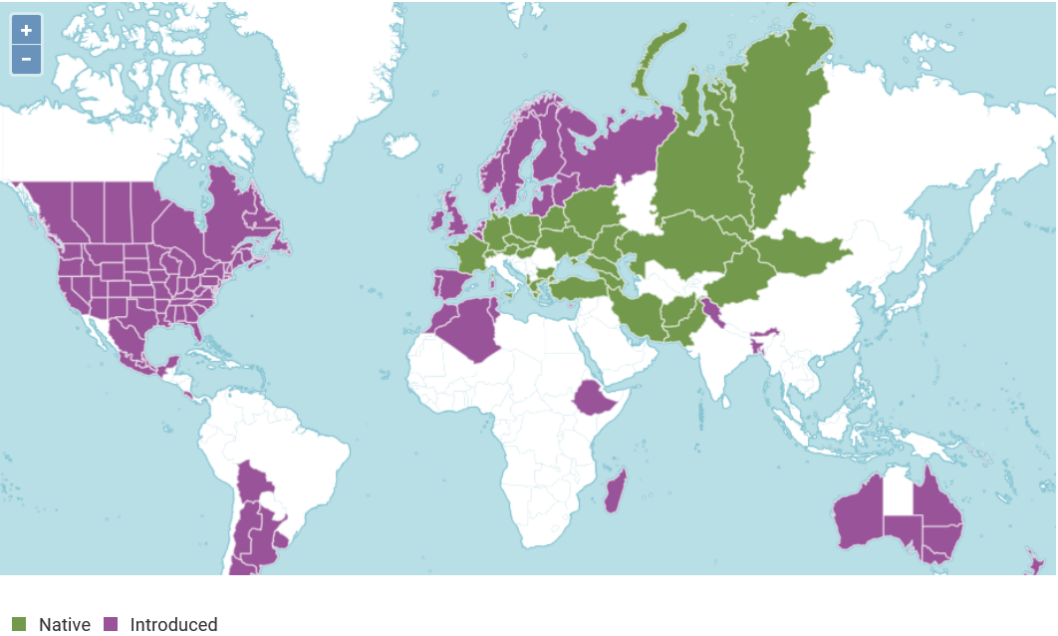
Distribution of cultivation of *Asparagus officinalis* around the world. The origin is believed to be eastern Mediterranean, however, it grows also in central Europe, the Caucasus and western Asia. It was brought centuries ago to North America, Northern Europe and parts of South America, North Africa and Australia. © Copyright 2017 World Checklist of Selected Plant Families. http://creativecommons.org/licenses/by/3.0 [3].

Asparagus *(A. officinalis)* is predominantly a food crop, solely eaten in the form of its very young thickened shoots, called spears. However, it and its related species have also been, and are still used as a source of medicinal bioactives. The crop has been cultivated and harvested for thousands of years by many ancient civilizations, including the Egyptians, ancient Greeks and Romans and so the crop has a rich and varied past. Its appearance as an offering, illustrated on an old Egyptian frieze, is a first indication of its use, although it is not clear whether the plant had a food or medicinal application at the time. There are many references to its use in ancient Greek and Roman times both as a food and as a source of herbal medicine. It is known to have been grown in French monasteries in the mid-15th century but apparently only arrived a century later in Germany and England. Introduction to North America occurred much later, in the mid-19th century. Today, it is widely consumed right across the world. Furthermore, its potential healing powers are still recognised in Traditional Chinese Medicine practices [1]. Taxonomically, when the monocot family *Liliaceae* was divided, Asparagus became a member of the new family *Asparagaceae* [2]. *A. officinalis* is only one of many *Asparagus* spp. and is an herbaceous perennial native to (Northern) Europe, NW Africa and is found in Asia as far east as Mongolia. However, through farming/escapes the species is now widespread as a (persistent) weed in a much broader range of countries including those in N. America, Scandinavia and Australia [Figure 1].

**Figure 2:**
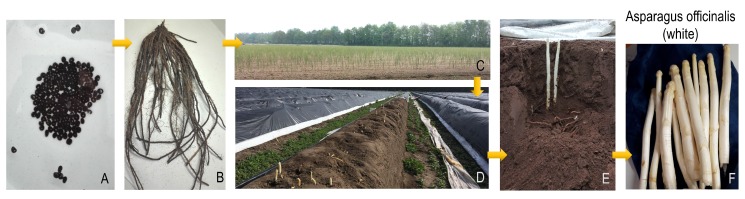
*Asparagus officinalis* (white) from seed to harvested product. (**A**) asparagus seeds, primed in water in order to help germination when sown. (**B**) asparagus root crown, one-year old, ready to be planted in the field. (**C**) new asparagus field with two-year old plants. (**D**) asparagus field in harvesting season with plastic covers to raise soil temperature. The black plastic covers the plants during the whole harvesting period to eliminate light exposure to the spears. (**E**) white asparagus shoots emerging from 40 cm deep in the soil. (**F**) harvested white asparagus spears. All pictures taken in Limburg, The Netherlands (spring 2019).

Successful production of the asparagus crop requires strict cultivation conditions, such as a specific soil composition, good drainage and the correct temperature range. Seeds are germinated and the plants are grown for one year before being transplanted to the final production location with the correct agronomic features [Figure 2A–C]. Plants must then be allowed to grow there for at least a second year in order to develop a strong root crown for the coming year’s production [Figure 2B,C]. From the third year on, spears can be harvested for consumption [Figure 2D–F]. The commercial production period can last for up to 10–12 years after which the plants should be discarded and the field used for the cultivation of a crop other than asparagus. Harvesting of the emerging spears can only be done for a specific period as the plant must then be allowed to produce a leafy crown to build up photosynthetic reserves to survive through the following winter and produce the next year’s spears. In Europe, the season is usually from late-April until mid-June but earlier harvesting is becoming possible through either greenhouse production or even by warming the soil below the roots using a modified central heating system. Harvesting too late or using incorrect storage conditions can result in inedible woody spears [4,5,6].

The asparagus crop recognises two main types—green (and green–purple) and white. Some countries (The Netherlands, Belgium, Peru) are more familiar with/favour the white varieties, while others (e.g., the UK) usually only see the green form in supermarkets. The green form traditionally has a much bigger global market to the extent that some countries only see this form and consumers may not even be aware of the existence of the white variant. This has led to some scientific papers not even mentioning which form was used in the research (so one has to assume that it is green). Agronomically, the key difference is whether the shoots are harvested above ground (green) or underground before they reach the surface/light (white) [Figure 2]. Botanically, both types are a single species and while, in some cases, the same variety can be used to produce both variants, breeders have chosen to breed for varieties which are better suited to one of the two production methods. Asparagus is a rather challenging crop requiring intense manual labour efforts during the short harvesting period lasting 7–8 weeks in the spring. However, once harvesting stops, the plants still require extensive care while the produced foliage provides the required sugars through photosynthesis for storage in the roots until die-back in the early autumn. While asparagus was considered for many years as a luxury/gourmet food item, the crop has become more broadly available and more widely eaten in recent years. For example, production in Europe almost doubled to more than 300,000 tonnes/year between 1968 and 2017 [7]. However, the time and cost needed to establish, manage and manually harvest asparagus fields, as well as the crop’s strong seasonality limited to just the first few weeks of growth, leads to the high price of the crop. This is likely to keep asparagus in the most expensive vegetable category at least until production methods require less input. Automated harvesting machines for white asparagus are currently being tested and if successful, might lead to a major change in the industry. Nevertheless, in 2017 the crop was already in the top 20 most eaten vegetables in both the USA and Europe [8]. China leads global production (ca. 57,000 Ha in 2018; mostly green). In N. America, in the USA and Mexico, respectively, 23,000 and 22,000 Ha are grown (mostly green) and in S. America, Peru leads the way with 20,000 Ha (mostly white). In Europe, total production covers ca. 65,000 Ha with Germany (both white and green) being by far the biggest grower with 22,000 Ha— twice the No. 2 (Spain) with 11,000 Ha [9,10]. The most recent market predictions indicate that the global asparagus market will continue to grow by ca. 3% per year to reach a global market volume of ca. 10 million metric tonnes by 2027 with a total market value of 37 Bn US$ [11,12].

Considering the importance of the crop, its growing global market across all continents, its value as a nutritious flavoursome food and its potential healing properties (see further below), there is a clear growing interest in gaining deeper knowledge of asparagus and its composition. Asparagus is considered high in basic nutrients, including vitamins, minerals and amino acids [13,14,15,16] and is a fibre-rich food [15,17,18] [see Table 1]. However, despite its long history, there is relatively little knowledge of the crop and especially of its chemistry and its impact on human physiology after ingestion. Flavour and fragrance are key food quality traits which for asparagus, have only infrequently been the subject of detailed study.

In this short review we present an overview of our current knowledge of the crop and describe our advancing need for new information. While we shall occasionally touch on the non-food aspects, the focus shall mainly be placed on the vegetable market for which most data are available. We shall also address how modern scientific approaches such as metabolomics may help us gain deeper knowledge and allow us to design new strategies for developing a crop with improved quality and which meets future consumer needs.

## 2. Asparagus Biochemistry

The genus *Asparagus* consists of almost 300 species [23]. In the past, roots of some species were used by herbalists for their various properties, e.g., *Asparagus curilus* for diabetes and dysentery [24], *Asparagus filicinus* for rheumatism [25] and *Asparagus racemosus* for epilepsy, night blindness and hypercholesteremia [26,27] while *Asparagus officinalis* aqueous root extracts have been associated with the regulation of main reproductive hormones and oogenesis in mammals [28]. These various properties attributed to asparagus would seem to underline a complex and interesting biochemistry. Studies on the phytonutrients of asparagus have shown a rich mixture, including saponins, flavonoids and other phenolics [23,29]. In this section, an overview of the current knowledge of the biochemical composition of *Asparagus officinalis* is given in relation to the main groups of compounds discovered.

### 2.1. Steroidal Saponins

Saponins are high molecular-weight glycosides consisting of a conjugate of an oligosaccharide to a triterpene or a steroid aglycone. They naturally occur in plants and have often been associated with medicinal properties [30]. The aglycone of a steroidal saponin is usually a spirostanol or a furostanol [31]. Steroidal saponins play an important role in the biological and pharmacological activities of different *Asparagus* species [23,32,33]. The main saponins present in most of the green and white commercial varieties *(Asparagus officinalis)* are asparanin A [34], protodioscin [32,34,35,36,37], sarsasapogenin [38,39] and yamogenin [38,40] [see Table S1]. Interestingly the saponin profiles of other *Asparagus* spp., such as *A. maritimus* and *A. prostrates* which are from southern Europe, were different than the one from *A. officinalis*, where indeed protodioscin was the main saponin [41]. Investigating the properties of all detected saponins in different species and varieties of asparagus might suggest alternative uses of the non-food *Asparagus* spp. Studies on asparagus saponins have revealed in vivo their hypolipidemic effects due to their contribution in decreasing LDL and total cholesterol levels, improving the health state of rats that were fed a high-cholesterol diet [42,43]. Saponins extracted from asparagus shoots have also been associated with having antitumor [34,39,44,45,46,47] and antifungal [33,48] effects in vitro. Apart from such important biological activities, these compounds are also invaluable for the characteristic bitter taste of asparagus [49,50,51,52] as described further below.

### 2.2. Vitamins

Asparagus is a valuable nutritional source of several vitamins. In Table 1, the levels of important vitamins that are present in 100 g of asparagus (raw and cooked) are listed. In the top-14 healthiest vegetables worldwide, asparagus ranks 10th [19], and in Table 1, it is shown that the levels of B vitamins do not deviate much from the No. 1 vegetable in the top-14. B vitamins, especially vitamin B6, vitamin B12 and vitamin B9, contribute to maintaining a healthy level of homocysteine in blood. Elevated levels of homocysteine increase the risk of atherosclerosis and other cardiac disorders [53]. Furthermore, folate, next to maintaining healthy levels of homocysteine in the body, through conversion to methionine, plays a crucial role in cell division and the formation of DNA [54]. The high folate content of asparagus shoots emphasizes the added nutritional value of the vegetable and the advantage of its consumption especially, for example, during pregnancy [55,56]. Traces of vitamin B12 are also found in asparagus and this vitamin is essential for general cell physiology and is a co-factor in the effective biosynthesis of DNA, amino acids and fatty acids [57]. Asparagus also contains smaller amounts of vitamin K and vitamin E, which also contribute to a healthy diet.

### 2.3. Minerals

Many essential minerals are present in asparagus. These include selenium, iron, calcium, copper, zinc, magnesium, potassium and phosphorus [see Table 1]. The content of these minerals was studied as a function of the position within the spears for two asparagus varieties and significant spatial variation was observed [58]. Minerals were found to be most concentrated in the upper sections of the spears, close to the tip. This indicates a gradient of chemistry in asparagus which is discussed further in later parts of this review. In Table 1, the recommended daily intake value of all nutrients is given, showing how much an excellent source of minerals asparagus is. Levels presented entail that a reasonable asparagus consumption (a full portion of asparagus corresponds to 180 g) makes a valuable contribution to the dietary intake of these essential minerals.

### 2.4. Flavonoids and Other Phenols

Flavonoids and other phenolic compounds have been the focal point of many analyses of asparagus materials [see Table S1], motivated by their potential antioxidant and anticarcinogenic properties [59]. Evaluation of these properties was performed through the analyses of the total phenols [60,61,62] or specific phenolic subclasses such as flavonoids, comparing different varieties and/or cultivation systems [63,64]. Fuentes-Alventosa analysed 32 commercial hybrids of green asparagus and 65 genotypes of the Spanish asparagus variety *Triguero*, which is a wild green-purple asparagus consumed in Southern Spain [63]. Clustering analysis revealed that the flavonoid profiles of some *Triguero* genotypes are significantly different from the American commercial hybrids that were analysed. This is of potential importance as Vazquez-Castilla et al. were able to show that the flavonoid content of *Triguero* spears was one of the characteristics linked to improving plasma lipid and liver antioxidant status in hypercholesterolemic rats [43]. As an example of one important flavonoid in asparagus, the quantification of rutin (quercetin-3-O-rutinoside), revealed that this flavonoid is present in the upper sections of green asparagus spears (1.51–7.29 mg/g dry weight) [35] explaining a possible correlation between rutin content and the green asparagus tissues that are exposed longer to sunlight. Next to this, traces of rutin have also been detected in white asparagus (below 0.5 mg/g dry weight), but still following a concentration gradient with highest levels in the upper parts. Earlier data showed similar results when comparing the content of rutin in different parts of (assuming green) asparagus [36]. Rutin was detected in the upper sections at levels 0.03–0.06% fresh weight, but also in the lower parts at levels below 0.01% fresh weight. Both studies [35,36] imply a concentration gradient as also seen for minerals. Rutin, together with 93 additional compounds (of which 32 phenolic compounds), was also detected in green asparagus shoots in an untargeted analysis by Jimenez-Sanchez et al. who performed Reversed Phase High Performance Liquid Chromatography Electro Spray Ionization Quadrupole Time-of-Flight Tandem Mass Spectrometry $\left(RP-HPLC-ESI-QTOF/MS^{2}\right)$ for profiling metabolites in green asparagus and identified 74 compounds that were not reported before for asparagus [60]. This list included hydroxycinnamic acids, which play a role in the cell wall biochemistry of asparagus shoots [65]. These studies highlight the various roles that phenolics might have in the complex biochemistry of asparagus.

### 2.5. Volatile Sulphur Compounds and Their Precursors

Sulphur-containing compounds (S-compounds) have a prominent role in plants and their derived products, relating to plant protection aspects [66], to human health-promoting activities [67,68] and to flavour and fragrance attributes [69]. S-compounds form a rather interesting compound class in asparagus that have attracted the attention of several studies that we discuss and refer to in this review. Asparagusic acid (1,2-dithiolane-4-carboxylic acid) is an S-compound which has been reported as being unique in asparagus [70,71] and has been of great interest concerning both pharmacological [70,72] and flavour properties [50,71,73] of asparagus. All reported S-compounds in asparagus materials are listed in Table S2. A number of S-compounds were found to contribute to the typical asparagus flavour. These include dimethyl sulphide (DMS), methanethiol and methional [70,71,73,74]. From these S-volatiles, DMS is considered the main key odorant in cooked asparagus [74] and the fact that it is formed upon cooking/processing means that its precursor, S-methylmethionine [75] is an equally crucial compound in the vegetable. Recently, another novel S-compound, asparaptine has been discovered and detected in both green and white asparagus spears, and shown to have an inhibitory activity against the Angiotensin-Converting Enzyme (ACE), in vitro [76]. In a completely different context, some S-compounds have also been highlighted as being major contributors to the characteristic urine odour following asparagus consumption [77]. However, there is still a lot of confusion as to whether these are the main odorants contributing to the smell, or if there are other compounds derived from several biosynthetic pathways [70,77,78]. This particular topic is described in more detail at the end of this article. Considering the importance of volatile compounds to various consumer-related quality attributes, exploring the biochemical pathways of the S-volatiles in asparagus in more detail is needed to help shed more light on the origins of these important compounds, also in the context of food quality and health [67].

## 3. Cultivation, Harvesting, and Storage Influences on Asparagus Quality

Asparagus cultivation is a rather challenging process that requires commitment and dedication as well as patience and flexibility to adapt to the varying weather conditions which can highly influence the productivity and final crop quality of the asparagus field. Testing the soil composition is crucial to determine the nutrient needs both for the new and the established fields. Based on soil type, different concentrations of phosphorus, potassium and trace elements are recommended for the healthy growth of asparagus roots, shoots and ferns. Careful nutrient applications during the harvest years ensures for continued healthy spears and high productivity. After 10–12 years of harvesting, an asparagus field is cleared and the field prepared for the cultivation of another crop. It is advised not to continue harvesting spears for much longer than a decade from the same field, because as well as lower harvest yields and thinner spears, there is an increasing chance that the spears become infected by pathogens. The main fungal pathogens infecting asparagus are *Fusarium* spp. which cause the roots to rot [79,80,81]. Breeders are concerned and engaged in the development of resistant varieties, as it is not possible to directly control *Fusarium* spp. and the best approach is to prevent the initial infection [82].

Concerning the productivity of asparagus, another factor is the sex of the plants. There are productive differences between male and female asparagus plants that make the male plants sprout earlier and produce both larger and more spears per rootstock weight than the female plants do [83,84]. Consequently, asparagus growers prefer male plants, and due to the indistinguishable morphological differences between the two sexes at early stages of growth [83], attempts to differentiate males from females based on seedling phenotype [85] and molecular markers [86,87,88,89] have kept breeders busy for a long time. These studies have allowed researchers to construct genetic maps that help in the identification of sex chromosomes in *A. officinalis* [90,91] and to exploit asparagus as a model plant for studying the origin of sex chromosomes [92].

Focusing back to the method of cultivation, evaluation of different cultivation systems of asparagus by multivariate statistics showed separation between conventionally and organically grown spears based on analysed bioactive compounds [64]. Furthermore, also in this study, comparing different parts of the crop (cladodes and spears), showed that different tissues have significantly different compositions of the bioactive polyphenolics. Another recent study, aiming to correlate taste differences among different spear sections of asparagus, showed that the content of phenolic and organic acids was dependent on the position within the spear (increasing from base to top) [93]. Consequently, these results, in combination with similar conclusions concerning mineral concentrations [58], imply a differential profile of metabolites within the asparagus shoot which is developmentally regulated. However, it is not clear how this might be further affected by environmental conditions present during growth and storage. In addition, while genetic factors together with the environment evidently influence the final quality of the crop, the (phyto)chemical composition and thus the potential health of the plants can also be affected by microbes in the environment in both a positive and negative way. For example, in the case of *A. officinalis*, the profile of natural headspace volatiles was shown to vary between healthy and black cutworm-induced stems [94]. Consequently, studies focusing specifically on genotype variation should also bear in mind the influence of both abiotic and biotic stresses in these analyses.

Cultivation practice and the moment of harvesting (e.g., soil, temperature, environmental conditions at harvest) may influence productivity [95] and the final quality of crop. These quality differences have, for example, been correlated with levels of specific compound classes, such as the phenolics and flavonoids, in connection to their antioxidant capacity [64,96]. Based on the USDA Agricultural Marketing standards, the basic attributes for asparagus grading are length, diameter and colour uniformity. However, considering the ever-increasing demands of the modern consumer, we would propose that more attention be given to additional consumer-relevant characteristics such as flavour as described previously [97,98,99,100].

Harvesting has a direct impact on the metabolism of plant materials, as it can lead to the activation of plant defence and stress mechanisms and eventually senescence and decay, leading to the production of several protective secondary (volatile and non-volatile) metabolites and proteins [101]. The impact of cultivation conditions, harvesting and storage of asparagus on its antioxidant capacity have been thoroughly examined [14,64,65,96,102,103]. In white asparagus, so far the focus has been on the total phenolic content [96] and specifically, ferulic acid derivatives [65,102]. Here, it was shown that an increased level of ferulic acids is associated with post-harvest spear hardening [65], and that post-harvest storage temperature plays an important role in cell wall hardening [4,6,102,103]. Other studies have shown that storage temperatures above 10 °C can have significant impact on both the appearance and the texture of the spears [104]. Such indications imply the high perishability of asparagus meaning a relatively short shelf life, that short farm-to-shop times are essential and strictly controlled conditions are required for maintaining the quality of the crop after harvest. The whole topic of the influence of environmental, genetic and post-harvest storage factors on asparagus quality has been recently well reviewed by [105] who again stressed the need for a better understanding of the influence of environmental factors specifically on sensory-active phytochemicals.

Cold storage of asparagus spears does not completely deactivate all biochemical mechanisms that might play a role in final crop quality, including texture [4]. The accumulation of ferulic acid, a hydroxycinnamic acid, has been verified to increase in the cell walls of asparagus under different storage conditions and contributes to the hardening of the spears [65,102]. In this hardening process the content of the polysaccharide heteroxylan in the spear cell walls also increases, which has made asparagus a plant model also for studying the heteroxylan biosynthesis [5]. Concerning the colour of the spears, an increase in anthocyanins in white asparagus was reported upon post-harvest light-exposure leading to a slight purple colorization [104]. This also entails a reduction in quality/market value. Therefore, a conscious understanding of the biochemical background of asparagus is needed in order to define the important determinants influencing the quality of the vegetable and, therefore the optimal post-harvest handling strategy.

## 4. Asparagus and Health

A significant daily fruit and vegetable consumption is considered essential for a healthy diet as promoted by the ‘5 per day’ campaign running in the USA, UK and other European countries and on the basis of WHO advice [106]. Apart from nutritionists and health professionals, a growing audience is tending to pay ever-growing attention to the potential health benefits and properties of especially the vegetables and fruits that are consumed, as well as how they are prepared. Being rich in vitamins and antioxidants (see above) and with a high fibre and relatively low-calorie content [see Table 1], consuming *A. officinalis* spears makes an excellent contribution to a healthy diet. Furthermore, its mineral content [Table 1] makes it of value to specific patient types such as those with hypertension [107] considering the potassium content of asparagus. However, this and other *Asparagus spp*. have also been linked to more medicinally based applications in the form of herbal medicines and here we also briefly provide an overview of these non-food applications.

Traditionally, *Asparagus* spp. have been especially used in China and Korea, as a source of herbal medicines. For example, thanks to its diuretic properties, the vegetable has found application for the treatment of urinary problems [1,13,23]. In India, asparagus root extracts have been used to strengthen the female reproductive system, promote fertility and increase breast milk production [1]. In both ancient Eastern and Greek medicine, asparagus extracts have been used as a tonic for the prevention and cure of several ailments including those for the kidney, bladder, rheumatic, liver disease, asthma and cancer [1,13,108,109]. Nevertheless, despite these long-term traditional medicinal uses, such applications have never been FDA-approved and hence, pharmacological use beyond those as ‘traditional medicines’ has not gained wide acceptance due to insufficient convincing clinical evidence. However, new studies using more advanced approaches to find the potential bioactive components using proper combinations of cell-based assays and clinical trials may contribute to the discovery of novel medicines from *Asparagus* spp. in the global quest for new pharmaceuticals.

Being a rich source of phytochemicals with antioxidant attributes has highlighted asparagus shoots as being a valuable dietary component [23,61,110]. Asparagus spear extracts have been correlated to suppressive effect on elevated blood glucose in type 2 diabetic rats [111]. Fan et al. compared different extraction solvents and methods focusing on the antioxidant compounds in asparagus waste materials using HPLC-MS/MS [62]. They detected ferulic acid, rutin, kaempferol, quercetin and isorhamnetin in the analysed samples, without mentioning whether they were detected as aglycones or glucosides. All of these compounds had been identified earlier in the context of the high antioxidant status of the crop [36,60] and they have been promoted for their potentially health-boosting characteristics [112]. As mentioned above, another main class of chemical constituents in asparagus is the steroidal saponins, which were of great interest in a number of earlier studies [32,45,48]. Next to the reported antitumor properties of asparagus saponins, it was suggested that there are also antifungal properties of the saponin which was first reported as yamoscin [48]. The structure of this saponin is 3−O−[α−L−rhamnopyranosyl(1→2)α−L−rhamnopyranosyl(1→4)−β−D−glucopyranosyl](25s)spirost−5−ene−3−β−ol which is likely also the saponin named Yamogenin II [40]. More recent studies have taken advantage to expand on these data and enrich our knowledge of the potential anticancer properties of asparagus steroidal compounds [39,47]. The reported saponin was isolated from the lower parts of white asparagus spears which are generally discarded as waste [48]. This together with the analysis of Fan et al. [62] represent possibly interesting bioresource application opportunities for the crop.

There are also health-related aspects regarding the group of S-compounds for which asparagus has become well known (see above). Organic compounds such as isothiocyanates, allicin, and sulphides that are present in garlic, onion (*Allium* spp.) and broccoli (*Brassica oleracea*) are already being applied to help decrease LDL (bad cholesterol) and blood pressure, and in preventing cancer [113,114]. *Asparagus* spp. have indeed already been associated with hypocholesterolemic effects [26,42,43]. As discussed above, in asparagus the central sulphur compound is asparagusic acid. Derivatives of asparagusic acid, together with some other sulphur compounds from asparagus were observed to inhibit cyclooxygenase 2 (COX-2) activity, which is an inducible enzyme associated with inflammatory diseases and carcinogenesis [72]. A targeted metabolomics approach for S-compounds, using LC-Fourier Transform Ion Cyclotron Resonance (FTICR) MS, detected a new S-compound in asparagus spears, which they called asparaptine $\left(C_{10}H_{19}N_{4}O_{3}S_{2}\right)$, and contains the same 1,2-diothiolane ring present in asparagusic acid. Asparaptine has been proposed to have inhibitory activity against the angiotensin-converting enzyme (ACE), which plays a role in hypertension regulation in humans [76,115]. These findings might imply that asparaptine shall gain a crucial role for the pharmacological image of asparagus.

Despite there still being no reported human trials to verify the many potential health benefits of asparagus consumption, or the compounds listed above, interest in asparagus phytochemicals is growing. References to traditional medicine applications and consideration of the aforementioned studies on potential health benefits of different compound groups present, place asparagus again in the spotlight as a potential source of novel bioactives for which the pharmaceutical industry is urgently searching.

## 5. Asparagus Flavour

Asparagus flavour can be divisive; many love the delicate bitter complexity, while others are revolted from what can seem a strange vegetal tang. The typical asparagus flavour plays a central role in its promotion. The so-called ‘white gold’ (white asparagus) is usually considered to have a milder and more delicate taste than its green counterpart. Being considered a delicacy of the vegetable world, when in season (end-April till mid-June in Europe) asparagus provides the main ingredient in several dishes, both in haute cuisine as well as in traditional food dishes. Asparagus is often reported with aroma descriptions similar to grassy white wines such as unoaked Sauvignon Blanc [116]. Other equivalent wine descriptors that have been linked to asparagus include fresh, savoury and bitter senses [117]. These experiences are accredited to specific volatile compound classes and in particular to aldehydes, pyrazines and sulphur compounds. The intensity balance of these compounds can affect how appealing, or not, the overall flavour is to an individual consumer. While as yet there are few publications on asparagus flavour, a start has been made. A summarized list, based on the literature, of the main key odorants in cooked asparagus is presented in Table 2. The associated perceived aroma attributes have also been added where these are known, together with the odour thresholds of these compounds in water. Based on these figures, clear differences in potential flavour impact are observed. However, odour thresholds are dependent on the type of food matrix [118,119] and this must be taken into consideration in any evaluation.

Flavour can be studied both in a descriptive and an analytical manner. The descriptive way corresponds to the results of human sensory trials, where trained panellists assess the flavour, in the form of aroma and/or taste and texture of a food product [119,120]. The analytical route refers to the techniques used to obtain the metabolite profile of the food product of interest and the two might be joined to some extend by exploiting Gas Chromatography Olfactory (GC-O) approaches. Which metabolites are observed depends not only on which are actually present in the sample but also which specific extraction, separation and detection methods will be used for the analysis. Metabolomics tools are nowadays increasingly being applied for both qualitative and quantitative analysis of food materials [121]. Non-volatile compounds that contribute to the taste, such as semi-polar secondary metabolites, are usually analysed using NMR (Nuclear Magnetic Resonance) or LC-MS methods, while volatile compounds that contribute to the aroma are analysed using GC-MS techniques [122]. However, the application of comprehensive (untargeted) metabolomics approaches have hardly yet been exploited for asparagus. Analytical chemistry studies on the asparagus flavour date from four decades ago [73,74,99,123,124]. Combinations of analytical techniques with sensory panels have also been used with the aim to unravel the aroma and taste of asparagus in terms of individual chemicals and to correlate these to cultivars and environmental factors [73,74,98,124], as well as to processing techniques [125]. However, most of the studies performed have tended to focus on a limited pre-defined list of compounds, which have been proposed to be the key odorants. This list, reported here in Table 2, contains C6 alcohols and aldehydes and other volatile organic compounds derived from the fatty acid degradation pathway, as well as some S-compounds and pyrazines which may be formed during cooking/processing, due to heat treatment. As a result, such an approach may be biased in driving conclusions repeatedly to existing known compounds and runs the risk of misinformation. This might better be tackled with more advanced approaches using initially an untargeted more holistic analytical methodology.

**Figure 3:**
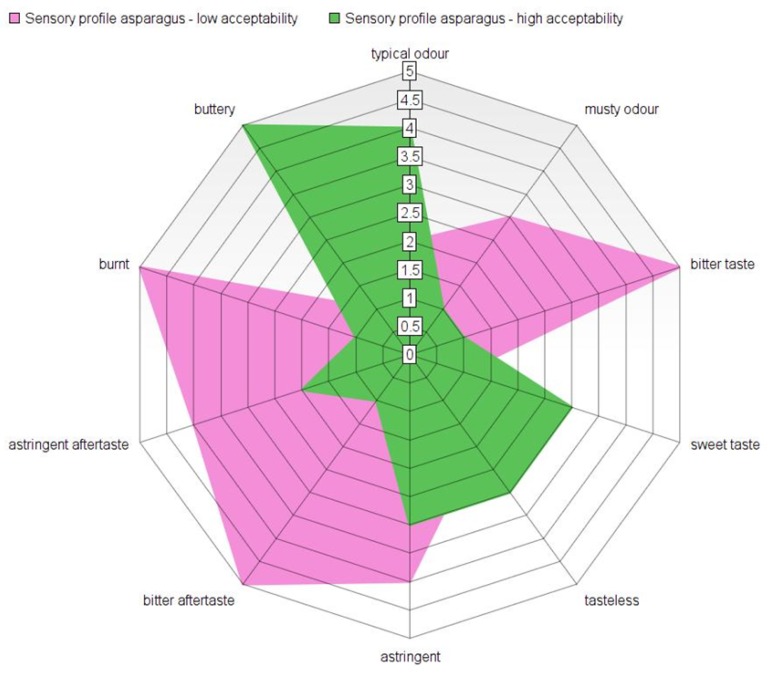
Re-worked summary of the data from [97].The sensory profile of asparagus with low (pink) and high (green) acceptability as was calculated based on the average of the sensory profiles of 12 different cultivars in Europe in 2004. The typical odour corresponds to the right balance of compounds and aroma attributes presented in Table 2 and Figure 4. The musty odour is basically due to unbalanced ratios of C8 ketones and alcohols, based on the findings of [128].

The final flavour of asparagus, as of any vegetable, is a rather complex combination of aroma and taste. The levels of bitterness seem to be a crucial characteristic for acceptance by consumers [98,99,127]. Bitter-tasting saponins (mainly furostanol saponins [51,52]) likely play an important role here and interestingly, different saponins are responsible for the bitter taste in fresh and in cooked asparagus [49,50]. Hoberg and Ulrich proposed what might be the right balance between sweetness and bitterness, as based on European consumer preference [97]. Figure 3 represents a re-worked summary of the data from that study where it was recommended that breeders and producers should increase the sweetness and reduce bitterness in white asparagus cultivars for an improved consumer sensory experience. However, flavour perception and assessment are highly dependable on societal habit and local culture. Therefore, any such conclusions should take into consideration the season as well as the geographical location/social custom of the reported analysis. For the clearest picture, sensory evaluations should be combined with analytical assessments, in order to allow us to build up the most detailed picture and to correlate specific (groups of) compounds with specific sensory experiences.

**Figure 4:**
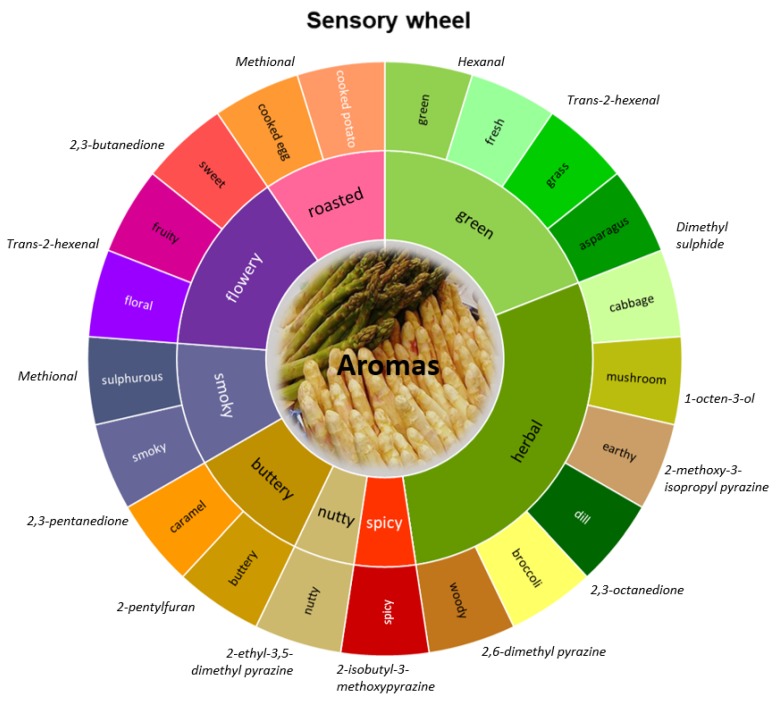
The Asparagus Sensory Wheel - constructed based on key odorants and sensory attributes from the literature reported in this review. The key odorants from Table 2 are included next to the main aroma attribute of their contribution for the typical asparagus odour. The key to a pleasant aroma profile is the right balance of all the aroma attributes and therefore, concentrations of the key odorants.

All in all, asparagus flavour is a complex delicacy for those who appreciate it. The different aroma notes that it comprises are the key to its uniqueness. Based on the literature and the aroma attributes of specific volatile compounds, we constructed the sensory wheel shown in Figure 4. This can be seen as the Asparagus Sensory Wheel, summarizing all the aromas, the right balance of which creates the characteristic cooked asparagus aroma (the “typical odour” as indicated in Figure 3).

## 6. Asparagus and the Smelly Urinary Story—A Catalogue of Misconceptions

One of the more noteworthy features of eating asparagus is the often observed and indeed, discussed consequence - in the form of the distinctly odorous urine of the consumer. Indeed, human physiology seems to go into overtime as the rapidity of this occurrence is remarkable in that this can be noticeable within just a few minutes. Our growing understanding of this process makes for an interesting story of physiological, sensory and genetical intrigue.

Table 3 gives a brief potted history of key seminal moments in this smelly urine story. The phenomenon of odorous urine was already observed long ago, already being noted by Lemery in 1702 [129] and later also referred to by many prominent authors and polymaths such as Arbuthnot [130], Benjamin Franklin [131] and Proust [132]. Only later did it become clear that not everyone actually produces smelly urine and furthermore, not everyone can smell it. These first reports from around the mid-18th century have interestingly been proposed to be linked to a change in agronomic practice at that time related to the start of using inorganic and organic S-containing fertilizers to boost plant yield [133]. ‘Recognition of smelly urine appears coincidental with the start of the use of S containing fertilizers’ [77] and this relevance of sulphur quickly became clear once the first chemical investigations were carried out.

Looking into which components were potentially causal to asparagus-associated urine odour, in 1891 Nencki first reported a potential link to the presence of one S-compound, methanethiol [134]. Later results were subsequently questioned as it was considered that the extraction/detection methods used might induce the appearance of artefacts rather than reveal the true in vivo compounds [133]. Later however, growing numbers of S-compounds were reported [137,144]. Waring, using GC-MS, detected 6 S-compounds, including the originally reported methanethiol, as well as dimethyl sulphide—a highly volatile, low odour threshold compound [133]. Furthermore, both these compounds were also confirmed using trained panellists and standard compound solutions as ‘having a smell reminiscent of asparagus urine odour’. In 2001, Leitner reported in total 12 volatile S-compounds detected in asparagus urine, several of which also have low odour thresholds [140]. Summarising the literature to date, Pelchat in 2011 provided the most extensive table of sulphur-containing odorants (n = 29) so far found in Asparagus urine [142]. These results strongly suggest that while S-compounds are potentially causal, it is also likely that the urine odour is the result of a complex mixture rather than a single component. The origin of these S-compounds is still a cause of speculation. Many of these odorants are not found in fresh asparagus [133] and also not after cooking (where indeed, their highly volatile nature would likely lead to their loss) [73]. This infers that they arise as the result of human metabolism working on chemical precursors, perhaps through the digestion of compounds such as S-methylmethionine and asparagusic acid [71,133]. Interestingly, the latter is considered to be unique to asparagus and is proposed as the ‘ most probable culprit’ as the source of the specific asparagus urine odour [70]. A theoritical set of chemical conversions from asparagusic acid to the observed odorants has been proposed as being feasible but no evidence has yet been provided [73,77]. To elucidate this further we might consider a combination of an untargeted GC-based metabolomics approach coupled to two detection methods in parallel. A Mass Spectrometer would give in depth chemical information on those compounds present and a second detector—the human nose, in the form of a GC-O analysis using a sniffing port, would help us link individual compounds to bioactivities (odour). In this way, we would not only get an unbiased overview of the chemical mixture, but we could reveal any potential links between individual components and their sensory relevance.

More than half a century ago it was recognized and reported for the first time that the phenomenon of humans producing smelly urine is actually not universal [135]. Allison & McWhirter reported a polymorphism within 115 individuals for the ability to produce and excrete odorous methanethiol after asparagus consumption, with the excreter genotype being dominant [136]. Mitchel reported that just 43% out of 800 volunteers were ‘excreters’ after asparagus consumption [139]. In 1980, from a study of 328 Israelis of different ethnic backgrounds it was concluded that some people could not smell asparagus urine and that ca. 10% were the opposite, being ‘hypersensitive’ and still able to detect the odour after significant dilution [138]. This phenomenon of anosma (smell blindness) is a recognized genetically determined trait [138] as is hyperosmia or an over-sensitivity for certain odour compounds. Although there may be some ethnical relationships [77] it is evident that the global population comprises both excreters and non-excreters as well as smellers and non-smellers and indeed, in all possible combinations. Eriksson performed the first 23andMe web-based analysis using voluntary information obtained from 10,000 subjects on the ability to smell ‘asparagus urine’ [141]. Despite failing to recognize the complication that many volunteers in the cohort will have been in the ‘smeller but non-excreter’ category and hence will give false negative information, the analysis did allow the identification of a genetic link to a region of Chromosome 1. Later, but taking into account the smeller/excreter complexity issues, Pelchat was able to confirm the Eriksson finding and also concluded that the abilities to excrete/smell appear not to be genetically linked [142]. A later study by Markt et al. remarkedly also failed to recognize the complexity of this odour phenomenon but again reported 50-60% of individuals were anosmic and polymorphic for an SNP in the same Chromosome 1 region where the OR2 olfactory receptors are located as was previously identified [143].

So, to conclude this historical investigation which is still ongoing, the central current dogma is that firstly, almost certainly not all asparagus varieties produce the same amounts of substrate, and secondly, while certain individuals can secrete the smelly compounds but not smell them, others can smell the compounds despite not making them themselves and there are those that do both or neither. Thirdly, there also seems to be quantitative variation in both abilities.

## 7. The Potential of Asparagus Metabolomics

Asparagus is a crop which has long been at the centre of attention due to its unique cultivation requirements, its typical flavour and its health-promoting potential. For *A. officinalis* there are many reports of not only its characteristic unique flavour [49,50,73,74,98,99,123,124] but also its medicinal properties [1,13,23,33,46,47,48,61,62,76,108,109]. All of these features, as well as visible differences between e.g., varieties, cultivation strategy etc. are directly related to the biochemical composition of the spears. However, our knowledge is still rather limited despite the importance of the crop on a global scale. Untargeted metabolomics approaches [145,146] could effectively be exploited to this purpose as these have great potential to advance our chemical knowledge and help us unravel the complex biochemical pathways in asparagus, covering both primary and secondary (volatile and non-volatile) metabolites. Plant metabolomics can contribute by exposing the complex physiology and biochemistry of plants as reported before [147]. Furthermore, MS-based imaging (MSI) techniques such as laser ablated electrospray ionisation (Laser Ablation Electrospray Ionization LAESI; [148,149] and Matrix assisted laser desorption/ionization – imaging MS (MALDI-IMS) [150], can provide additional useful information on the localization of metabolites in planta. Untargeted metabolomics approaches have already successfully been applied to investigate the metabolome of for instance eggplant fruits [151], tomato fruit [152], potato tubers [153] and coffee leaves [154], providing useful information for breeders and manufacturers. A combination of robust metabolomics technologies for analysing primary and both volatile and non-volatile secondary metabolites in asparagus will contribute in expanding and improving our knowledge of the crop.

For asparagus, most analytical chemical analyses carried out to date have generally been focused on specific (sub)groups of metabolites and have failed to reveal the true chemical complexity of the materials. This is also evident for example, by examining the currently used metabolite databases: searching the key word “asparagus” in the current largest food metabolite database (FooDB [155]) retrieves just 211 compounds. In the KNAPSACK-DataBase [156] 74 compounds are registered and the Dictionary of Natural Products [157] includes 194. Clearly, many more metabolites must be present but have yet to be identified and registered and metabolomics can play an important role here. The first true untargeted metabolomics experiments are underway and indeed, even more advanced technologies are being considered in our quest to establish deep knowledge of asparagus biochemistry. Recently, *A. officinalis* spears (green) were used in a proof-of-concept study, applying a newly developed tissue sectioning protocol to visualize and locate specific metabolites within plant tissues using MALDI-IMS [150,158]. Here, MALDI-IMS allowed the localization of asparaptine (recently detected as a new compound [76]) and revealed another metabolite “gradients” in the spear adding to those from previous studies covering minerals and phenolics. Nakabayashi also focused on flavonoids and correlation analysis revealed the flavonoid rutin to co-localize with a number of other metabolites across the different tissues analysed, suggesting, a common biochemical regulation [150]. The same study also showed a lower accumulation of rutin in those spear tissues not exposed to sunlight. This potentially contradicts previous results [35] where rutin was found to be more predominant in the upper spear sections than in lower ones. MS-based high-resolution imaging methodology (like MALDI-IMS) offers an additional spatial dimension for metabolite analysis and, together with complementary, comprehensive metabolomics approaches will help reveal the detailed metabolome of asparagus in both spatial and temporal terms. To our knowledge, an untargeted (unbiased) metabolomics approach, aiming to detect and compare as many metabolites present in asparagus as possible has not yet been applied. Furthermore, few biochemical pathways related to quality traits in asparagus have been fully unravelled.

## 8. General Conclusions

Asparagus is a major global crop of growing importance. Several *Asparagus* spp. have been used for their medicinal properties [24,25,26,27] but nowadays, asparagus is mainly used and valued as a vegetable. Appreciation from consumers depends on certain quality parameters mainly focused on appearance. Included here are colour uniformity, the length and thickness of the spear and the tendency of the head to split making the spear unsellable. However, important consumer-relevant quality parameters such as aroma and taste have largely been ignored by breeders. This is mainly because we still have only a meagre understanding of which chemical factors determine these phenotypic attributes and of their underlying biochemical pathways. Furthermore, how these quality parameters are genetically and environmentally influenced also remains largely a black box. However, some studies have led to the proposal that there are certain key odorants (specific volatile compounds) that are considered mainly responsible for the typical asparagus flavour. However, the exact importance and contribution of each of these components still requires clarification. Exploiting unbiased, comprehensive metabolomics approaches might take us some way further in defining the complex biochemistry of this crop and identifying all the key compounds which underlie sensory quality and other crop properties.

## Figures and Tables

**Table 1:** Nutritional overview of raw and cooked asparagus (100 g) and for reference, the Recommended Daily Intake (RDI) of the nutrients based on a 2000 kcal diet. Indicated are also the values of the listed nutrients present in spinach considered as the No. 1 of the 14 healthiest vegetables in the world [19].

Nutrient	Value per 100 g Raw Asparagus [20,21] (10th in the List of Top-14 Healthiest Vegetables)	Value per 100 g Cooked Asparagus [22]	Value per 100 g Raw Spinach (1st in the Top-14 Healthiest Vegetables)	RDI based on a 2000 Kcal Diet
Overal Nutrition Facts				
Calories	20 kcal	20 kcal	23 kcal	2000 kcal
Dietary fibres	2.1 g	2 g	2.2 g	25 g
Sugars	1.9 g	1.3 g	0.4 g	90 g
Proteins	2.2 g	2.4 g	2.9 g	40–50 g
Fat content	0.12 g	0.22 g	0.4 g	65 g
Vitamins				
Vitamin B1, thiamin	0.143 mg	0.162 mg	0.078 mg	1.4 mg
Vitamin B2. riboflavin	0.141 mg	0.139 mg	0.189 mg	1.6 mg
Vitamin B3, niacin	0.978 mg	1.1 mg	0.724 mg	15 mg
Vitamin B9, folate	52 μg	149 μg	194 μg	400 μg
Vitamin C, ascorbic acid	5.6 mg	7.7 mg	28.1 mg	75 mg
Vitamin E, alpha-tocopherol	1.13 mg	1.5 mg	2.03 mg	10 mg
Vitamin K	41.6 μg	50.6 μg	482.9 μg	80 μg
Minerals				
Calcium, Ca	24 mg	23 mg	99 mg	1000 mg
Copper, Cu	0.19 mg	0.19 mg	0.13 mg	0.9 mg
Iron, Fe	2.14 mg	0.91 mg	2.71 mg	15 mg
Magnesium, Mg	14 mg	14 mg	79 mg	350 mg
Manganese, Mn	0.158 mg	0.158 mg	0.897 mg	5 mg
Potassium, K	202 mg	224 mg	558 mg	3500 mg
Selenium, Se	2.3 μg	10.8 μg	1 μg	35 μg
Sodium, Na	2 mg	14 mg	79 mg	1500 mg
Zinc, Zn	0.54 mg	0.54 mg	0.53 mg	15 mg

**Table 2:** Summarized list of the key odorants in cooked asparagus flavour, based on the literature. The concentration in asparagus, odour threshold in water [126] and the characteristic aroma attribute of each volatile are also indicated if known (MW: molecular weight in g/mol, nq: not quantified, nd: not determined).

Volatile Compound, Molecular Formula (MW)	Reported Concentration in Asparagus (ppb)	Odour Threshold in Water (ppb)	Aroma Attribute	Reference
dimethyl sulphide, $C_{2}H_{6}S$ (62.14)	3300	0.12	Sulphurous, onion-like, asparagus	[73,74,123,127]
2,3-butanedione, $C_{4}H_{6}O_{2}$ (86.09)	nq	8.6	Sweet, buttery, caramel	[73,74]
3-methylthio-propionanal, $C_{4}H_{8}OS$ (104.17)	nq	0.2	Sulphurous, cheesy, cooked egg, baked potato	[74,127]
2,3-pentanedione, $C_{5}H_{8}O_{2}$ (100.12)	nq	20	Buttery, caramel, roasted, nutty	[73,74,127]
trans-2-hexenal, $C_{6}H_{10}O$ (98.14)	13	17	Green, fresh, fruity	[73,74,123,127]
hexanal, $C_{6}H_{12}O$ (100.16)	100–260	4.5	Green, fresh, grass, woody	[73,74,123,127]
2,6-dimethyl pyrazine, $C_{6}H_{8}N_{2}$ (108.14)	200	800–1800	Earthy, rusty, nutty, woody, greasy	[73,74,127]
2-ethyl-3,5-dimethyl pyrazine, $C_{8}H_{12}N_{2}$ (136.19)	nq	1	Nutty, roasted, coffee	[73,74,127]
2-methoxy-3-isopropyl pyrazine, $C_{8}H_{12}N_{2}O$ (152.19)	nq	0.002–10	Earthy	[73,74]
2,3-octanedione, $C_{8}H_{14}O_{2}$ (142.2)	nq	nd	Cooked, buttery, dill-like, broccoli-like	[73,74,127]
1-octen-3-ol, $C_{8}H_{16}O$ (128.21)	42–300	1	Earthy, mushroom-like	[73,74,98,123,127]
2-isobutyl-3-methoxypyrazine, $C_{9}H_{14}N_{2}O$ (162.22)	nq	0.002–0.016	Spicy, earthy, green, sprout-like	[74,127]
2-pentylfuran, $C_{9}H_{14}O$ (138.21)	2–165	6	Buttery, earthy	[73,74]

**Table 3:** Seminal moments in the history of smelly asparagus urine.

Year	Observation	Reference
Literature recognition		
1702	Asparagus.... causes a powerful/filthy and disagreeable smell in the urine as everybody knows.	[129]
1731	“ ....Of the Stems of Plants, some contain a sine Aperient Salt, and are Diaretick and Saponaceous, as Asparagus which affects the Urine with a Fetid Smell (especially if cut when they are white)....” (- and later included in the definition of Asparagus in Samuel Johnstons First Dictionary of the English Language Vol 1 Edition 1 (1755))	[130]
1770	“A few Stems of Asparagus eaten, shall give our Urine a disagreable Odour”	[131]
1913	"....but what fascinated me would be the asparagus, .... all night long after a dinner at which I had partaken of them, they played .... at transforming my humble chamber into a bower of aromatic perfume."	[132]
Scientific		
1891	Urine smell proposed to be related to S-compound first identified as methanethiol	[134]
1956	Polymorphism reported within 115 individuals—two genes assoc-iated with ability to produce/excrete the S-compound (methane-thiol) in urine after eating Asparagus; excretor gene is dominant	[135,136]
1975	Adding two S-compounds to urine resulted in the characteristic odour ‘likely through formation of methanethiol’	[137]
1980	328 Israelis divided into smellers/non-smellers but concluded that production was ‘universal’ and suggest also a genetically determined odour hypersensitivity in 10% of volunteers.	[138]
1987	800 volunteers eat asparagus – just under half 43% are excreters. Family studies also confirmed genetic polymorphism/Autosomal dominant gene and phenotype not age or sex related	[139]
2001	12 S-compounds identified (many with low odour thresholds) using SPME GC-MS and excretion dynamics followed 10 min–16 h	[140]
2010	First web-based GWAS survey of asparagus eaters. 63% smelled; But failed to recognise that there may be non-producers/smellers. Appears to stem from a single switched base-pair mutation in a cluster of 50 genes coding for olfactory receptors	[141]
2011	GWAs but solved issue limiting conclusions from Eriksson related to discriminating non-producers/smellers etc. Basis of inability to produce is still unknown but inability to smell is linked to an SNP in a 50 gene cluster on Chromosome 1.	[142]
2016	Confirmed results of Eriksson but again made the mistake of not discriminating non-producers from non-smellers.	[143]

## Supplementary Materials

The following are available online at https://www.mdpi.com/2218-1989/10/1/17/s1, Table S1: List of non-volatile secondary metabolites in *A. officinalis*, Table S2: List of volatile secondary metabolites in *A. officinalis*.
