# Supplementary material for: Green and White Asparagus (Asparagus officinalis): A Source of Developmental, Chemical and Urinary Intrigue
Source: Metabolites. 2019 Dec 25;10(1):17. doi: 10.3390/metabo10010017 (PMC7022954; doi:10.3390/metabo10010017)
Supplement: Supplementary file 1 [file metabolites-10-00017-s001.pdf]

metabolites

Supplementary Material

Green and white asparagus (*Asparagus officinalis*): A Source of Developmental, Chemical and Urinary Intrigue

Eirini Pegiou [1], Roland Mumm [2], Parag Acharya [3], Ric C.H. de Vos [2], Robert D. Hall [1,2,4,*]

[1] Wageningen University & Research, Laboratory of Plant Physiology, P.O. Box 16, 6700AA Wageningen, The Netherlands; eirini.pegiou@wur.nl
[2] Wageningen University & Research, Business Unit Bioscience, P.O. Box 16, 6700AA Wageningen, The Netherlands; roland.mumm@wur.nl (R.M.); ric.devos@wur.nl (R.C.H.d.V.)
[3] Unilever Foods Innovation Centre, Bronland 14, 6708WH Wageningen, The Netherlands; parag.acharya@unilever.com
[4] Netherlands metabolomics Centre, Einsteinweg 55, 2333CC Leiden, The Netherlands
* Correspondence: robert.hall@wur.nl

Supplementary Tables

Table S1: List of non-volatile secondary metabolites detected and reported in *A. officinalis* (green and/or white) materials (when not indicated, this was not reported in the article and green can be assumed). For the description of the analytical techniques the following codes apply; the separation or ionization technique (HPLC: high performance liquid chromatography, MALDI: matrix assisted laser desorption/ionization) and the detector (MS: mass spectrometry, UV: ultraviolet, NMR: nuclear magnetic resonance, I: imaging) (MW: molecular weight in g/mol, FW: fresh weight, DW: dry weight).

Detected secondary metabolite, Molecular formula (MW)	*Asparagus material (reported concentration)*	*Analytical technique (Reference)*
	Polyphenols	
Flavonoids		
quercetin-3-O-rutinoside (rutin), $C_{27}H_{30}O_{16}$ (610.5)	Green spears (2.28 mg/100 g FW and 0.05 % FW in top section) [1]	HPLC-MS [1–4]
	Green spears (ca. 7 mg/g DW in top section, 5 mg/g DW in middle section, 2 mg/g DW in bottom sections)	HPLC-UV at 210 nm [5]
	White spears (<0.5 mg/g DW)	HPLC-UV at 210 nm [5]
	White spears (1.1 mg/100 g FW) & green spears (48.2 mg/100 g FW)	HPLC-MS [6]
	Cooked white spears	HPLC-MS [7]

	Asparagus waste	HPLC-UV & MS [8]
	Green spears	HPLC-MS [9] and HPLC-MS/MS & MALDI-IMS [10]
apigenin-6-8-di-C-glucoside (vicenin 2), $C_{27}H_{30}O_{15}$ (549.5)	Green spears	HPLC-MS [9]
apigenin rhamnoside, $C_{21}H_{20}O_{9}$ (416.4)		HPLC-MS [3]
quercetin-3-O-glucosyl-rutinoside, $C_{33}H_{40}O_{21}$ (772.7)		HPLC-MS [9]
quercetin-3-β-D glucoside, $C_{21}H_{19}O_{12}$ (463.4)		HPLC-MS [3,9]
quercetin diglucoside, $C_{27}H_{30}O_{17}$ (626.5)		HPLC-MS [9]
quercetin triglycoside, $C_{33}H_{40}O_{22}$ (788.7)		HPLC-MS [4]
quercetin galactoside, $C_{21}H_{20}O_{12}$ (464.38)		HPLC-MS [3]
isorhamnetin-3-O-rutinoside, $C_{28}H_{32}O_{16}$ (624.5)		HPLC-MS [3]
isorhamnetin-3-O-rutinoside (narcissin), $C_{28}H_{32}O_{16}$ (624.5)		HPLC-MS [4,9]
noricaritin-O-glucoside (amurensin), $C_{26}H_{30}O_{12}$ (534.5)		HPLC-MS [9]
kaempferol-3-O-rutinoside (nicotiflorin), $C_{27}H_{30}O_{15}$ (594.5)		HPLC-MS [4,9]
kaempferol glucoside,	Green spears	HPLC-MS [3,9]
$C_{21}H_{20}O_{11}$ (448.4)	Asparagus waste	HPLC-UV & MS [8]
kaempferol-O-acetylglucoside, $C_{23}H_{22}O_{12}$ (490.4)	Green spears	HPLC-MS [9]
kaempferol-3-p-coumaroyl-glucoside,		

$C_{30}H_{26}O_{13}$ (594.52)		
quercetin, $C_{15}H_{10}O_7$ (302.24)	Green spears (0.14 mg/100 g FW)	HPLC-MS [2,9]
	Asparagus waste	HPLC-UV & MS [8]
Phenolic acids		
ferulic acid, $C_{10}H_{10}O_4$ (194.18)	Green spears (1.05 mg/100 g FW)	HPLC-MS [2,3,9]
	Asparagus waste	HPLC-UV & MS [8]
	Cooked white spears	HPLC-MS [7]
vanillic acid, $C_8H_8O_4$ (168.15)	Green spears	HPLC-MS [9]
3-caffeoylquinic acid (chlorogenic acid), $C_{16}H_{18}O_9$ (354.31)		HPLC-MS [3,9]
3-p-coumaroylquinic acid, $C_{16}H_{18}O_8$ (338.31)		HPLC-MS [3,9]
5-caffeoylquinic acid (neo-chlorogenic acid)/1-caffeoylquinic acid, $C_{16}H_{18}O_9$ (354.31)		
Saponins		
protodioscin, $C_{51}H_{84}O_{22}$ (1049.2)	Asparagus seeds	1H- & 13C-NMR [11]
	Asparagus spears (0.24 mg/100 g FW in top section, 25mg/100 g FW in bottom section)	HPLC-MS [1]
	Green spears (<1 mg/g DW) White spears (ca 2.6 mg/g DW in top section, 3.7 mg/g DW in middle section, 4-5 mg/g DW in bottom section)	HPLC-UV at 210 nm [5]
	White spears (0.66-13.6 mg/100 g) & green spears (0.3 mg/100 g)	HPLC-MS/MS [12]
dioscin, $C_{45}H_{72}O_{16}$ (869.05)	White spears (6.18-33.9 mg/100 g) & green spears (6.49 mg/100 g)	HPLC-MS/MS [12]
methyl protodioscin, $C_{52}H_{86}O_{22}$ (1063.2)	Green spears (0.65 mg/100 g)	
asparasaponin (ASP) I, $C_{51}H_{84}O_{22}$ (1049.2)	White spears (0.32-3.75 mg/100 g) & green spears (0.3 mg/100 g)	

asparasaponin II, $C_{45}H_{74}O_{18}$ (903.1)		
(25R/S)-dihydro-ASP-II	White spears (0.02-0.4 mg/100 g) & green spears (0.1 mg/100 g)	
yamogenin, $C_{27}H_{42}O_3$ (414.6)	White spears (bottom parts)	HPLC-UV at 210 nm [13]
	Green spears	HPLC-MS and 1H & 13C NMR [14]
sarsasapogenin, $C_{27}H_{44}O_3$ (416.6)	Asparagus roots	HPLC-MS and 1H & 13C NMR [15]
	Sulphur compounds	
asparagusic acid, $C_4H_6O_2S_2$ (150.2)	White spears (10.6 mg/100 g FW) & green spears (9.86 mg/100 g FW)	HPLC-MS [6,9]
	Green spears	HPLC-MS [9]
asparaptine, $C_{10}H_{18}N_4O_3S_2$ (306.4)	White spears (29.8 mg/100 g FW) & green spears (25.1 mg/100 g FW)	HPLC-MS [6]
	Green spears	HPLC-MS/MS & MALDI-IMS [16]
	Other compounds	
(1 stilbene, 2 lignans, 36 oxylipins, 2 glyceroglycolipids and 4 others)	Green spears	HPLC-MS [9]

Table S2: List of volatile secondary metabolites detected and reported in *A. officinalis* (green and/or white) materials (when not indicated, this was not reported in the article and green can be assumed). For the description of the analytical techniques the following codes apply; the trapping or extraction technique (SPE: liquid solid phase extraction, LLE: liquid liquid extraction, SDE: simultaneous steam distillation extraction, SPME: solid phase microextraction, TDU: thermal desorption), separation (GC: gas chromatography) and the detector (MS: mass spectrometry, O: olfactometry). (MW: molecular weight in g/mol). **key odorants based on the literature reported in this review and summarized in Table 2.

Detected secondary metabolites, Molecular formula (MW)	***Asparagus material (reported concentration in ppb)***	***Analytical technique (Reference)***
	Alcohols	
n-butanol, $C4H10O$ (74.12)	Cooked white spears (160)	SPE-GC-MS [17]

1-penten-3-ol, *C*5*H*10*O* (86.13)	Cooked white spears (80)	
2-penten-1-ol, *C*5*H*10*O* (86.13)	Cooked white spears (100)	
1-pentanol, *C*5*H*12*O* (88.15)	Cooked white spears (2300)	
	Cooked white spears	SDE-GC-MS [7,18–20]
2-pentanol, *C*5*H*12*O* (88.15)	Cooked white spears (25)	SPE-GC-MS [17]
2,4-hexadien-1-ol, *C*6*H*10*O* (98.145)	Cooked white spears (20)	
2-hexen-1-ol, *C*6*H*12*O* (100.16)	Cooked white spears (240)	
1-hexanol, *C*6*H*14*O* (102.162)	Cooked white spears (8400)	
	Cooked white spears	SDE-GC-MS [7,18,19]
2,4-heptadien-1-ol, *C*7*H*12*O* (112.17)	Cooked white spears (65)	SPE-GC-MS [17]
2-hepten-1-ol, *C*7*H*14*O* (114.186)	Cooked white spears (300)	
1-heptanol, *C*7*H*16*O* (116.88)	Cooked white spears (25)	
benzyl alcohol, *C*7*H*8*O* (108.14)	Cooked white spears (410)	
2-phenylethanol, *C*8*H*10*O* (122.16)	Cooked white spears (440)	
3-methyl-1-butanol, *C*5*H*12*O* (88.148)	Cooked white spears (80)	
1-octen-3-ol **, *C*8*H*16*O* (128.21)	Green asparagus juice (42.8)	SPME-GC-MS [21]
	Cooked white spears (300)	SPE-GC-MS [17]
	Cooked white spears	SDE-GC-MS [19,20]
	Asparagus spears	TDU-GC-MS [22]
2-octen-1-ol, *C*8*H*16*O* (128.21)	Cooked white spears (300)	SPE-GC-MS [17]
1-octanol, *C*8*H*18*O* (130.23)	Cooked white spears (70)	

2,4-nonadien-1-ol, $C_9H_{16}O$ (140.22)	Cooked white spears (50)	
2-nonen-1-ol, $C_9H_{18}O$ (142.24)	Cooked white spears (20)	
1-nonanol, $C_9H_{20}O$ (144.26)	Green asparagus juice (1)	SPME-GC-MS [21]
	Cooked white spears (60)	SPE-GC-MS [17]
2,4-decadien-1-ol, $C_{10}H_{18}O$ (154.249)	Cooked white spears (70)	
	Aldehydes	
2-butenal, C_4H_6O (70.09)	Green asparagus juice (25.5)	SPME-GC-MS [21]
	Cooked white spears	SDE-GC-MS [18–20]
3-methyl-butanal, $C_5H_{10}O$ (86.13)	Green asparagus juice (4.4)	SPME-GC-MS [21]
pentanal, $C_5H_{10}O$ (86.13)	Green asparagus juice (51.3)	SPME-GC-MS [21]
	Cooked white spears	SPE-GC-MS [17] and SDE-GC-MS [19,20]
2-methyl-2-butenal, C_5H_8O (84.12)	Green asparagus juice (31.3)	SPME-GC-MS [21]
2-pentenal, C_5H_8O (84.12)	Green asparagus juice (18.2)	
2-methyl-2-pentenal, $C_6H_{10}O$ (98.14)	Green asparagus juice (0.8)	
2-hexenal **, $C_6H_{10}O$ (98.14)	Green asparagus juice (13.2)	
	Cooked white spears	SPE-GC-MS [17] and SDE-GC-MS [18–20]
	Asparagus spears	TDU-GC-MS [22]
hexanal **, $C_6H_{12}O$ (100.16)	Green asparagus juice (259.2)	SPME-GC-MS [21]
	White spears (100)	SPE-GC-MS [17]
	Cooked white spears	SDE-GC-MS & GCO [7,19,20]

	Asparagus spears	TDU-GC-MS [22]
2,4-heptadienal, $C7IH10O$ (110.15)	Green asparagus juice (2)	SPME-GC-MS [21]
2-heptenal, $C7H12O$ (112.17)	Green asparagus juice (52.7)	
	White spears (30)	SPE-GC-MS [17]
	Cooked white spears	SDE-GC-MS [7,19,20]
heptanal, $C7H14O$ (114.188)	Green asparagus juice (1)	SPME-GC-MS [21]
benzaldehyde, $C7H6O$ (106.12)	Green asparagus juice (0.9)	SPME-GC-MS [21]
	Cooked white spears	SPE-GC-MS [17] and SDE-GC-MS [7]
2,4-octadienal, $C8H12O$ (124.18)	Green asparagus juice (1.6)	SPME-GC-MS [21]
2-octenal, $C8H14O$ (126.2)	Green asparagus juice (17.5)	
	Cooked white spears	SPE-GC-MS [17] and SDE-GC-MS [19,20]
octanal, $C8H16O$ (128.212)	Green asparagus juice (1.7)	SPME-GC-MS [21]
phenylacetaldehyde, $C8H8O$ (120.15)	Cooked white spears	SDE-GC-MS [18–20]
2,4-nonadienal, $C9H14O$ (138.21)		
2-nonenal, $C9H16O$ (140.226)	Cooked white spears (10)	SPE-GC-MS [17]
nonanal, $C9H18O$ (142.239)	Green asparagus juice (3.4)	SPME-GC-MS [21]
2-phenyl-2-butenal, $C10H10O$ (149.19)	Cooked white spears (10)	SPE-GC-MS [17]
2,4-decadienal, $C10H16O$ (152.23)	Green asparagus juice (1.1)	SPME-GC-MS [21]
	Cooked white spears	SPE-GC-MS [17] and SDE-GC-MS [7,19,20]

2-dodecenal, $C_{12}H_{22}O$ (182.3)	Green asparagus juice (1.1)	SPME-GC-MS [21]
	Ketones	
2,3-butanedione **, $C_4H_6O_2$ (86.09)	Cooked white spears	SDE-GC-MS [7,18–20]
3-hydroxy-2-butanone, $C_4H_8O_2$ (88.11)	Cooked white spears (6400)	SPE-GC-MS [17]
3-hydroxy-2-pentanone, $C_5H_{12}O_2$ (102.13)	Cooked white spears (80)	
2,3-pentanedione **, $C_5H_8O_2$ (100.12)	Cooked white spears	SDE-GC-MS & GCO [7,18–20]
2-hexanone, $C_6H_{12}O$ (100.161)	Cooked white spears (10)	SPE-GC-MS [17]
2,5-cyclohexadienone, C_6H_6O (94.11)	Cooked white spears (90)	
2-heptanone, $C_7H_{14}O$ (114.18)	Cooked white spears (15)	
3,5-octadien-2-one, $C_8H_{12}O$ (124.18)	Green asparagus juice (3.9)	SPME-GC-MS [21]
3,5-octadien-2-one, $C_8H_{12}O$ (124.18)	Cooked white spears (70)	SPE-GC-MS [17]
1-octen-3-one, $C_8H_{14}O$ (126.2)	Green asparagus juice (22.5)	SPME-GC-MS [21]
6-methyl-5-hepten-2-one, $C_8H_{14}O$ (126.2)	Green asparagus juice (1.5)	
3-octen-2-one, $C_8H_{14}O$ (126.2)	Green asparagus juice (0.7)	
3-octen-2-one, $C_8H_{14}O$ (126.2)	Cooked white spears (10)	SPE-GC-MS [17]
2,3-octanedione **, $C_8H_{14}O_2$ (142.2)	Cooked white spears	SDE-GC-MS & GCO [18–20]
3-octanone, $C_8H_{16}O$ (128.21)	Green asparagus juice (2.3)	SPME-GC-MS [21]
3,5-nonadien-2-one, $C_9H_{14}O$ (138.207)	Cooked white spears (10)	SPE-GC-MS [17]
3,5-decadien-2-one, $C_{10}H_{16}O$ (152.23)	Cooked white spears (10)	
β-ionone, $C_{13}H_{20}O$ (192.3)	Green asparagus juice (1.4)	SPME-GC-MS [21]

geranyl acetone, $C_{13}H_{22}O$ (194.31)	Green asparagus juice (0.3)	
Esters		
ethyl dodecanoate, $C_{14}H_{28}O_2$ (228.37)	Green asparagus juice (0.7)	SPME-GC-MS [21]
diisobutyl phthalate, $C_{16}H_{22}O_4$ (278.34)	Green asparagus juice (1.9)	
ethyl hexadecanoate, $C_{18}H_{36}O_2$ (284.5)	Green asparagus juice (0.6)	
Carboxylic acids		
3-methylbutanoic acid, $C_5H_{10}O_2$ (102.13)	Green asparagus juice (1)	SPME-GC-MS [21]
pentanoic acid, $C_5H_{10}O_2$ (102.13)	Green asparagus juice (0.6)	
hexanoic acid, $C_6H_{12}O_2$ (116.158)	Green asparagus juice (2.1)	
Pyrazines		
2-methylpyrazine, $C_5H_6N_2$ (94.11)	Cooked white spears	SDE-GC-MS [7,19,20]
2-ethylpyrazine, $C_6H_8N_2$ (108.14)	White spears	SPE-GC-MS [17]
2,5-dimethylpyrazine, $C_6H_8N_2$ (108.14)	White spears (35)	
	Cooked white spears	SDE-GC-MS [19,20]
2,6-dimethylpyrazine **, $C_6H_8N_2$ (108.14)	White spears (200)	SPE-GC-MS [17]
	Cooked white spears	SDE-GC-MS & GCO [7,19,20]
2,3-dimethylpyrazine, $C_6H_8N_2$ (108.14)	White spears	SPE-GC-MS [17]
2-ethyl-6-methylpyrazine, $C_7H_{10}N_2$ (122.17)		
2-ethyl-5-methylpyrazine, $C_7H_{10}N_2$ (122.17)		
2-ethyl-3,5-dimethyl pyrazine **, $C_8H_{12}N_2$ (136.19)	Cooked white spears	SDE-GC-MS & GCO [19,20]
2-methoxy-3-isopropyl pyrazine **, $C_8H_{12}N_2O$ (152.19)		

2-isobutyl-3-methoxypyrazine **, $C_9H_{14}N_2O$ (152.19)	Cooked white spears	SPE-GC-MS [17] & SDE-GC-MS [20]
	Furans	
2-ethyl-furan, C_6H_8O (96.13)	Green asparagus juice (0.2)	SPME-GC-MS [21]
	Cooked white spears	SPE-GC-MS [17]
	Cooked white spears	SDE-GC-MS [19,20]
2-pentyl-furan **, $C_9H_{14}O$ (138.21)	Green asparagus juice (2.3)	SPME-GC-MS [21]
	Cooked white spears (165)	SPE-GC-MS [17]
	Cooked white spears	SDE-GC-MS [7,19,20]
	Asparagus spears	TDU-GC-MS [22]
	Sulphur compounds	
dimethyl sulfone, $C_2H_6O_2S$ (94.13)	Cooked white spears (30)	SPE-GC-MS [17]
dimethyl sulfoxide, C_2H_6OS (78.13)	Cooked white spears	SDE-GC-MS [19,20]
dimethyl sulfide **, C_2H_6S (62.14)	Cooked white spears (3300)	SPE-GC-MS [17]
	Cooked white spears	SDE-GC-MS [19,20]
S-methyl thioacetate, C_3H_6OS (90.15)	Cooked white spears	SDE-GC-MS & GCO [19,20]
3-methylthiopropanol, $C_4H_{10}OS$ (106.19)	Cooked white spears (10)	SPE-GC-MS [17]
methyl propyl sulfide, $C_4H_{10}S$ (90.19)	Cooked white spears	SDE-GC-MS [18,19]
3-methylthio-propanal **, C_4H_8OS (104.17)		SPE-GC-MS & GCO [17]
		SDE-GC-MS [7,19,20]
thiophene-2-carbaldehyde, C_5H_4OS (112.15)	Cooked white spears (10)	SPE-GC-MS [17]
2-acetylthiazole, C_5H_5NOS (127.17)	Cooked white spears (10)	
	Cooked white spears	SDE-GC-MS [19,20]

2-hydroxymethylthiophene, C_5H_6OS (114.17)	Cooked white spears (20)	SPE-GC-MS [17]
methyl-1,2-dithiolane-4-carboxylate, $C_5H_8O_2S_2$ (164.28)	Cooked white spears (7000)	
ethyl-1,2-dithiolane-4-csarboxylate, $C_6H_{10}O_2S_2$ (178.27)	Cooked white spears (50)	
2-formyl-3-methylthiophene, C_6H_6OS (126.18)	Cooked white spears (70)	
3-vinyl-3,4-dihydro-1,2-dithiine, $C_6H_8S_2$ (144.26)	Cooked white spears (20)	
	Other compounds	
methoxy-phenyl-oxime, $C_8H_9NO_2$ (151.16)	Green asparagus juice (18.9)	SPME-GC-MS [21]
2,4-di-tert-amylphenol, $C_{16}H_{26}O$ (234.38)	Green asparagus juice (4.3)	

Reference

1. Wang, M.; Tadmor, Y.; Wu, Q.L.; Chin, C.K.; Garrison, S.A.; Simon, J.E. Quantification of protodioscin and rutin in asparagus shoots by LC/MS and HPLC methods. *J. Agric. Food Chem.* **2003**, *51*, 6132–6136.
2. Fanasca, S.; Rouphael, Y.; Venneria, E.; Azzini, E.; Durazzo, A.; Maiani, G. Antioxidant properties of raw and cooked spears of green asparagus cultivars. *Int. J. Food Sci. Tech.* **2009**, *44*, 1017–1023.
3. Slatnar, A.; Petkovsek, M.M.; Stampar, F.; Veberic, R.; Horvat, J.; Jakse, M.; Sircelj, H. Game of Tones: sugars, organic acids, and phenolics in green and purple asparagus (Asparagus officinalis L.) cultivars. *Turk. J. Agric. For.* **2018**, *42*, 55–66.
4. Fuentes-Alventosa, J.; Jaramillo, S.; Rodriguez-Gutierrez, G.; Cermeño, P.; Espejo, J.; Jiménez-Araujo, A.; Guillén-Bejarano, R.; Fernández-Bolaños, J.; Rodríguez-Arcos, R. Flavonoid profile of green asparagus genotypes. *J. Agric. Food Chem.* **2008**, *56*, 6977–6984.
5. Lee, E.J.; Yoo, K.S.; Patil, B.S. Development of a rapid HPLC-UV method for simultaneous quantification of protodioscin and rutin in white and green asparagus spears. *J. Food Sci.* **2010**, *75*, 703–709.
6. Nakabayashi, R.; Yang, Z.; Nishizawa, T.; Mori, T.; Saito, K. Top-down targeted metabolomics reveals a sulfur-containing metabolite with inhibitory activity against angiotensin-converting enzyme in Asparagus officinalis. *J. Nat. Prod.* **2015**, *78*, 1179–1183.
7. Hoberg, E.; Ulrich, D.; Standhardt, D.; Kecke, S. Evaluation of Asparagus officinalis L. flavour quality for breeding purposes. IX International Asparagus Symposium 479, 1997, pp. 135–142.
8. Fan, R.; Yuan, F.; Wang, N.; Gao, Y.; Huang, Y. Extraction and analysis of antioxidant compounds from the residues of Asparagus officinalis L. *J. Food Sci. Technol.* **2015**, *52*, 2690–2700.
9. Jiménez-Sánchez, C.; Lozano-Sánchez, J.; Rodríguez-Pérez, C.; Segura-Carretero, A.; Fernández-Gutiérrez, A. Comprehensive, untargeted, and qualitative RP-HPLC-ESI-QTOF/MS2 metabolite profiling of green asparagus (Asparagus officinalis). *J. Food Compos. Anal.* **2016**, *46*, 78–87.
10. Nakabayashi, R.; Hashimoto, K.; Toyooka, K.; Saito, K. Keeping the shape of plant tissue for visualizing metabolite features in segmentation and correlation analysis of imaging mass spectrometry in Asparagus officinalis. *Metabolomics* **2019**, *15*, 24.
11. Shao, Y.; Chin, C.K.; Ho, C.T.; Ma, W.; Garrison, S.A.; Huang, M.T. Anti-tumor activity of the crude saponins obtained from asparagus. *Cancer Lett.* **1996**, *104*, 31–36.
12. Dawid, C.; Hofmann, T. Quantitation and bitter taste contribution of saponins in fresh and cooked white asparagus (Asparagus officinalis L.). *Food Chem.* **2014**, *145*, 427–436.

13. Shimoyamada, M.; Suzuki, M.; Maruyama, M.; Watanabe, K. An antifungal saponin from white asparagus (Asparagus officinalis L) bottoms. *J. Sci. Food Agric.* **1996**, *72*, 430–434.
14. Sun, Z.; Huang, X.; Kong, L. A new steroidal saponin from the dried stems of Asparagus officinalis L. *Fitoterapia* **2010**, *81*, 210–213.
15. Huang, X.; Kong, L. Steroidal saponins from roots of Asparagus officinalis. *Steroids* **2006**, *71*, 171–176.
16. Miyoshi, K.; Enomoto, Y.; Fukusaki, E.; Shimma, S. Visualization of Asparaptine in Asparagus (Asparagus officinalis) using MALDI-IMS. *Anal. Sci.* **2018**, pp. 997-1001
17. Tressl, R.; Bahri, D.; Holzer, M.; Kossa, T. Formation of flavor components in asparagus. 2. Formation of flavor components in cooked asparagus. *J. Agric. Food Chem.* **1977**, *25*, 459–463.
18. Hoberg, E.; Ulrich, D.; Gottwald, J.; Rosen, A. Environmental Influences on the Sensory Quality of Asparagus officinalis L. International Conference on Quality in Chains. An Integrated View on Fruit and Vegetable Quality 604, 2003, pp. 395–401.
19. Ulrich, D.; Hoberg, E. Flavor analysis in asparagus breeding. X International Asparagus Symposium 589, 2001, pp. 281–287.
20. Ulrich, D.; Hoberg, E.; Bittner, T.; Engewald,W.; Meilchen, K. Contribution of volatile compounds to the flavor of cooked asparagus. *Eur. Food Res. Technol.* **2001**, *213*, 200–204.
21. Chen, X.; Qin, W.; Ma, L.; Xu, F.; Jin, P.; Zheng, Y. Effect of high pressure processing and thermal treatment on physicochemical parameters, antioxidant activity and volatile compounds of green asparagus juice. *LWT-Food Sci. Technol.* **2015**, *62*, 927–933.
22. Sun, R.; Wang, Y.; Chin, C.K.; Garrison, S. Volatile Compounds in Asparagus officinalis L. X International Asparagus Symposium 589, 2001, pp. 257–266.
